# The transcription factor MML4_D12 regulates fiber development through interplay with the WD40-repeat protein WDR in cotton

**DOI:** 10.1093/jxb/eraa104

**Published:** 2020-03-02

**Authors:** Yue Tian, Jingjing Du, Huaitong Wu, Xueying Guan, Weihang Chen, Yan Hu, Lei Fang, Linyun Ding, Menglin Li, Duofeng Yang, Qinli Yang, Tianzhen Zhang

**Affiliations:** 1 National Key Laboratory of Crop Genetics and Germplasm Enhancement, Cotton Research Institute, Nanjing Agricultural University, Nanjing, P. R. China; 2 Zhejiang Provincial Key Laboratory of Crop Genetic Resources, Institute of Crop Science, College of Agriculture and Biotechnology, Zhejiang University, Zhejiang, China; 3 Australian National University, Australia

**Keywords:** Complex, cotton, fiber development, GhMML4_D12, GhWDR, trichome

## Abstract

*In planta*, a vital regulatory complex, MYB–basic helix–loop–helix (bHLH)–WD40 (MBW), is involved in trichome development and synthesis of anthocyanin and proanthocyanin in Arabidopsis. Usually, WD40 proteins provide a scaffold for protein–protein interaction between MYB and bHLH proteins. Members of subgroup 9 of the R2R3 MYB transcription factors, which includes *MYBMIXTA-Like* (*MML*) genes important for plant cell differentiation, are unable to interact with bHLH. In this study, we report that a cotton (*Gossypium hirsutum*) seed trichome or lint fiber-related GhMML factor, GhMML4_D12, interacts with a diverged WD40 protein (GhWDR) in a process similar to but different from that of the MBW ternary complex involved in Arabidopsis trichome development. Amino acids 250–267 of GhMML4_D12 and the first and third WD40 repeat domains of GhWDR determine their interaction. *GhWDR* could rescue Arabidopsis *ttg1* to its wild type, confirming its orthologous function in trichome development. Our findings shed more light towards understanding the key role of the MML and WD40 families in plants and in the improvement of cotton fiber production.

## Teaser

The transcription factor GhMML4_D12 interacts directly with a diverged WD40 protein (GhWDR), similar to but different from the MYB–bHLH–WD40 complex, in regulation of trichome development in cotton.

## Introduction

MYB transcription factors, especially R2R3 MYB, have a diverse range of functions in different plant species (Stracke *et al.*, 2001). Among them, subgroup 9, which includes those encoded by *MIXTA* genes, are important for the specification and regulation of plant cellular differentiation (Perez-Rodriguez *et al.*, 2005; Scoville *et al.*, 2011; Oshima *et al.*, 2013; Wu *et al.*, 2018; Yan *et al.*, 2018). The first *MIXTA* gene, characterized in snapdragon (*Antirrhinum majus*), was found to control the development of conical cell shape in the petal epidermis (Noda *et al.*, 1994). It also appears to be capable of driving the initiation of conical epidermal cells from flat epidermal cells (Glover *et al.*, 1998). The MYBMIXTA-Like (MML) transcription factors MYB106 and MYB16 regulate epidermal cell morphology and cuticle development in Arabidopsis and *Torenia fournieri* (Baumann *et al.*, 2007; Oshima *et al.*, 2013). In *Artemisia annua*, interaction between AaHD8 and AaMIXTA1 has a role in the formation of glandular trichomes and is involved in cuticle development (Yan *et al.*, 2017; Shi *et al.*, 2018). The R2R3 MYB transcription factors can form an MYB–basic helix–loop–helix (bHLH)–WD40 (MBW) complex to modulate diverse biological processes such as trichome development, cell death, cell wall synthesis, hormone signaling, stamen development, and seed production (Ramsay and Glover, 2005; Schaart *et al.*, 2013; Xie *et al.*, 2016). However, the MML transcription factors lack the core amino acids that have been reported to play a decisive role in interactions with bHLH to form the MBW complex. How *MML* genes regulate the specification of plant cellular differentiation remains to be explored.

In allotetraploid cotton, transcription factors encoded by 10 *GhMML* homologous genes contain the signature protein motif AQWESARxxAExRLxRES. They are all expressed during fiber initiation in cultivated cotton, but have lower expression in fiberless mutants (Zhang *et al.*, 2015). Using a map-based cloning strategy, we isolated and identified *GhMML3* on chromosome A12 (*GhMML3_A12*), responsible for fuzz fiber production (Wan *et al.*, 2016), and *GhMML4* on chromosome D12 (*GhMML4_D12*), responsible for development of lint fiber (*Li*_*3*_), the largest source of natural textile in the world (Wu *et al.*, 2018). A mutation that occurred at the 500 bp site from cytosine (C) in the linted–fuzzless or naked seed mutant (n_2_NSM) to adenine (A) in the Xuzhou142 fuzzless–lintless mutant (XZ142FLM) has resulted in the production of a TAA stop codon and early termination of GhMML4_D12 translation (Wu *et al.*, 2018). However, the mechanisms of how these *GhMML* genes control cotton fiber development are still unknown.

**Fig. 1.**
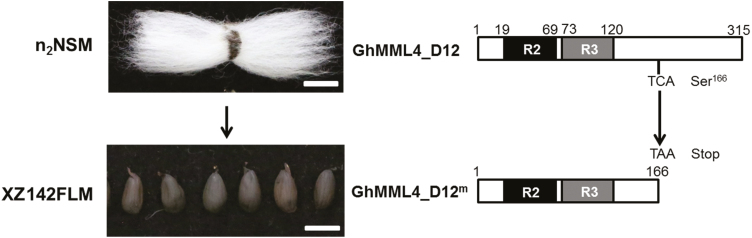
Phenotypic appearance of cotton fiber and structure of the *GhMML4_D12* gene between n_2_NSM and XZ142FLM. *GhMML4_D12* is from the linted–fuzzless accession (n_2_NSM) and the mutant *GhMML4_D12* gene (*GhMML4_D12*^*m*^) is derived from our Xuzhou142 fuzzless–lintless mutant (XZ142FLM). A mutation occurred at the 500 bp site, from cytosine (C) in n_2_NSM to adenine (A) in XZ142FLM, which resulted in the production of a TAA stop codon and early termination of GhMML4_D12 translation, Scale bar: 1.0 cm.

In this study, through a yeast two-hybrid (Y2H) assay, a new kind of WD40 repeat protein (GhWDR) was identified as an interacting partner of GhMML4_D12 from the naked seed mutant n_2_NSM, but not the mutant *GhMML4_D12* (*GhMML4_D12*^*m*^) derived from XZ142FLM (Fig. 1). We found for the first time a direct interaction between the MYB and WD40 transcription factors, and the GhMML4–GhWDR complex showed similarity to, but is different from, an MYB ternary complex. *GhWDR* could complement *ttg1* to its wild type, implying it has a conservative function in trichome development, and a WD40 repeat domain of GhWDR is important for its function in Arabidopsis. GhMML4_D12 could enhance its own transcriptional activity in n_2_NSM, but not in XZ142FLM.

## Methods

### Plant materials

*Gossypium hirsutum* acc. Texas Marker-1 (TM-1), the fuzzless or naked seed mutant n_2_NSM, and the fuzzless–lintless mutant XZ142FLM were used to investigate ﬁber development. All plants were grown at the Dangtu Breeding Station (DBS/NAU) with normal ﬁeld practices, and in the glasshouses of Nanjing Agriculture University. Arabidopsis seeds were sterilized with 20% bleach, plated on Murashige and Skoog medium (MS; Sigma-Aldrich), chilled at 4 °C for 3 d, and transferred to a growth room with a 16 h (22–24 °C)/8 h (19 °C) light/dark photoperiod. *Nicotiana benthamiana* was grown under a 16 h (28 °C)/8 h (22 °C) light/dark photoperiod.

### Quantitative reverse transcription polymerase chain reaction analysis

Total RNA was isolated from cotton tissues and purified using a Qiagen RNeasy kit (Qiagen) according to the manufacturer’s instructions. Expression profiling of genes in cotton tissues was carried out by quantitative reverse transcription polymerase chain reaction (qRT-PCR) using a cotton polyubiquitin gene, *GhUBI1* (EU604080), as a standard control according to a previously described method (Li *et al.*, 2005). In brief, cDNA was synthesized from total RNA and used as a template in qRT-PCR with gene-specific primers (see Supplementary Table S1 at *JXB* online). The PCR was performed using SYBR-Green Real-time PCR Master Mix according to the manufacturer’s instructions (Toyobo Co. Ltd, Osaka, Japan), and the relative quantity of the target gene expression level was determined. The mean value and standard deviation of three biological replicates were calculated.

### Subcellular localization of fusion protein

The full lengths of GhMML4_D12, GhMML4_D12^m^, GhWDR, N-terminal-truncated domain (GhWDR-N), WD40 repeat domain (GhWDR-R), and C-terminal-truncated domain (GhWDR-C) without a stop codon were inserted into the binary vector pBINGFP4 (Hellens *et al.*, 2000), upstream of the green fluorescent protein (GFP) sequence, to produce the constructs 35S_pro_:GhMML4_D12, 35S_pro_:GhMML4_D12^m^, 35S_pro_:GhWDR, 35S_pro_:GhWDR-N, 35S_pro_:GhWDR-R, and 35S_pro_:GhWDR-C. The constructs were introduced into *Agrobacterium tumefaciens* strain GV3101 by electroporation. Subcellular localization of all these fusion proteins was determined in tobacco (*Nicotiana benthamiana*) leaf epidermal cells by *A. tumefaciens* infiltration (Liu *et al.*, 2014). GFP fluorescence in the tobacco epidermal cell was observed using a Zeiss LSM 710 confocal microscope (Zeiss Microsystems) with a filter set of 466 nm for excitation and 506–538 nm for emission. Zen 2009 software (Zeiss) was used to record and process the digital images taken.

### Transcription activation analysis

To investigate the transcriptional activity of the GhWDR protein, the coding sequences (CDS) of GhWDR, GhWDR-N, GhWDR-R, and GhWDR-C were cloned into pGBKT7 vectors (Clontech, Palo Alto, CA, USA) and transferred into yeast strain AH109 using the high-efficiency lithium acetate transformation procedure. Yeast transformants were streaked on selective medium lacking tryptophan and adenine added to assay transcriptional activity. β-Galactosidase activity was also assayed by colony-lift ﬁlter assay using 5-bromo-4-chloro-3-indolyl β-D-galactopyranoside (X-gal) as the substrate.

### Phylogenetic analysis

Phylogenetic analysis was conducted using two methods: neighbor-joining (NJ) and maximum likelihood (ML). NJ trees were constructed using MEGA 5.1 (Tamura *et al.*, 2011) with 1000 bootstrap resampling and the Poisson correction model, plus the pairwise deletion option. RAXML v.8.2.4 was used to construct ML trees, with the Jones, Taylor, and Thorton (JTT) model, GTRGAMMA distribution option and 200 non-parametric bootstrap replicates (Stamatakis, 2014).

### Yeast two-hybrid screening and assays

A Y2H assay was performed using the Matchmaker GAL4 Two-Hybrid System following the manufacturer’s protocol (Clontech). Full-length cDNA of GhMML4_D12 was fused to the GAL4-DNA-binding domain of the bait vector pGBKT7 and transformed into yeast strain Y2H. A cDNA library from cotton ovules and fibers (−3 to ~25 d post-anthesis (DPA)) was constructed by transforming yeast strain AH109 with ds-cDNA and the pGADT7-Rec vector according to the manufacturer’s instructions. The library host strain was mated with bait strain Y2H, and the mating mixture was then spread onto SD/−Trp/−Leu/−His medium and incubated at 30 °C for 3~4 d. Positive clones were isolated and retransformed into bait strains to test their interaction using pGBKT7-p53/pGADT7 as the positive control and pGBKT7-Lamin c/pGADT7 as the negative control.

For the Y2H assay, all the GhMML4_D12 CDSs and their domain derivatives were cloned into pGBKT7, and the full-length or domain deletion forms of GhWDR were cloned into pGADT7. Primers used for the vector construction were presented in Supplementary Table S1. All the constructs were sequence verified. Self-activation of all the pGBKT7 fusion constructs was suppressed by different concentrations of Aureobasidin A (AbA). The indicated construct pairs were transformed into yeast strain Y2H and cultured at 30 °C. Successful yeast transformants were cultured in liquid SD minimal medium (−Trp/−Leu/−His) at 250 rpm and 30 °C for ~24 h to the final concentration (OD_600_=1.2–1.5). Then 1 ml of yeast strain was centrifuged and resuspended in distilled water to the final concentration (OD_600_=0.4–0.6). The mating mixture was diluted in multiples of 10 and 100, and then spread onto SD/−Trp/−Leu/−His medium with different concentrations of AbA and incubated at 30 °C for 3~4 d.

### Biomolecular fluorescence complementation

pSPYNE and pSPYCE plasmids have been reported previously (Waadt *et al.*, 2008). Full-length GhMML4_D12, GhMML4_D12^m^, and GhWDR were fused with the N- or C-terminal fragment of yellow fluorescent protein (nYFP or cYFP) to generate GhWDR-nYFP, cYFP-GhMML4_D12, and cYFP-GhMML4_D12^m^ plasmids. Primers used for plasmid construction are presented in Supplementary Table S1.

All constructs were transformed into *A. tumefaciens* strain GV3101. *Agrobacterium* strains containing different constructs were resuspended in infiltration buffer (10 mM MES, 0.2 mM acetosyringone, and 10 mM MgCl_2_) to a final concentration of OD_600_≈0.6–0.8. Equal volumes and concentrations of different combinations of *Agrobacterium* strains were co-infiltrated into *N. benthamiana* leaves. Plants were incubated at 23 °C for 2–3 d. YFP fluorescence was detected with a Zeiss microscope (Zeiss LSM710) and analysed using ZEN software. All experiments comprised three biological replicates.

### *In vitro* pull-down assays

The coding regions of GhMML4_D12 and GhMML4_D12^m^ were cloned into pET-GST, and the CDS of GhWDR was fused with pET-28a. These constructs were transformed into *E.coli* to express glutathione *S*-transferase (GST)–GhMML4_D12, GST–GhMML4_D12^m^, and His–GhWDR proteins, respectively. The pull-down assay was performed as described previously (Messina *et al.*, 2016). Briefly, 2 µg of purified fusion protein GST–GhMML4_D12 or GST–GhMML4_D12^m^ was incubated with immobilized 10 µg His–GhWDR fusion protein at 4 °C for 2 h and then separated by SDS-PAGE and immunoblotted with the corresponding antibody.

### Yeast one-hybrid assay

The CDSs of GhMML4_D12, GhMML4_D12^m^, and GhWDR were ligated into the pGADT7 vector (Clotech). *GhMML4_D12*, *GhMML4_D12*^*m*^, and *GhWDR* promoters were ligated into the pAbAi vector (Clotech). All primers used are listed in Supplementary Table S1. The yeast one-hybrid (Y1H) assay was conducted using the Matchmaker^TM^ Gold Yeast One-Hybrid Library Screening System kit (cat. no. 630491, Clontech, USA).

### Transient expression assay

A transient expression assay was performed in *N. benthamiana* leaves. The nuclear localization site (*NLS*) was ligated to the 3′ end of *GhMML4_D12* and *GhMML4_D12*^*m*^ promoters, then GFP was fused with the 3′ end of *NLS* to construct *GhMML4_D12*_*pro*_*:NLS-GFP* and *GhMML4_D12*^*m*^_*pro*_*:NLS-GFP*, respectively. The full length CDSs of GhMML4_D12, GhMML4_D12^m^, GhWDR, and GUS were driven by the *Cauliflower mosaic virus* (CaMV) 35S promoter to construct *35S*_*pro*_*:GhMML4_D12*, *35S*_*pro*_*:GhMML4_D12*^*m*^, *35S*_*pro*_*:GhWDR*, and *35S*_*pro*_*:GUS*. These constructs were then introduced into *A. tumefaciens* (strain GV3101). Infected tissues were analysed 48 h after inﬁltration. The GFP signal was observed under a confocal laser scanning microscope (Zeiss LSM710). All experiments were repeated with three independent biological replicates with similar results.

### Dual-luciferase assay

The *GhMML4_D12*, *GhMML4_D12*^*m*^, and *GhWDR* promoters were inserted into pGreenII 0800-LUC to drive the firefly LUC reporter gene, with *Renilla* (REN) luciferase controlled by the constitutive 35S promoter on the same plasmid as a reference to normalize infection efficiency. The CDSs of GhMML4_D12, GhMML4_D12^m^, and GhWDR were inserted into the pGreenII62-SK vector under the control of the 35S promoter. All primers used are listed in Supplementary Table S1. The constructs were transferred into *A. tumefaciens* (strain GV3101). The transformed *Agrobacterium* cells were mixed with the *Agrobacterium* strains harboring the effectors and reporters, in a volume ratio of 1:2.

Transient transformation was conducted by inﬁltration of the *Agrobacterium* mixtures into the abaxial side of *N. benthamiana* leaves using a syringe. After culturing for 3 d, ﬁreﬂy LUC and REN activities were measured using the Dual-Luciferase Reporter Assay System (E2490, Promega, USA), and the LUC/REN ratio was determined (the value of LUC was normalized to that of REN). Three biological repeats were measured for each combination.

### Complementation of Arabidopsis *ttg1* phenotypes

The full length CDS of the *GhWDR* were fused with pBINGFP4, under cotton native promoter. Binary construct (*GhWDR*_*pro*_*:GhWDR*) was introduced into *A. tumefaciens* strain GV3101 and subsequently transferred into Arabidopsis using the floral dip method (Clough and Bent, 1998). Selection of transformants was conducted on 0.8% agar containing Murashige and Skoog (MS) salts (2.2 g l^−1^) added with kanamycin (50 µg ml^−1^) for 7 d. Kanamycin-resistant seedlings were moved onto fresh kanamycin plates for 10 d before transfer to soil. Progeny from self-fertilized primary transformants were examined for complementation of other *ttg1* mutant phenotypes. Trichome restoration was examined by growing on plates containing MS salts and 1.2% phytagel for about 2 weeks. Root hair position was studied in seedlings grown vertically on plates containing MS salts and 1.2% phytagel for about 5–6 d. Anthocyanin synthesis was investigated in seedlings grown on plates containing MS salts and 1.2% phytagel for about 10 d. Seed coat color was examined by stereoscope after harvest.

*GhWDR-R* was fused with *GFP* driven by CaMV35S (*35S*_*pro*_*:GhWDR-R*) into *ttg1* Arabidopsis mutants. Selection of transformants and phenotype examination were as above.

## Results

### Molecular characterization of GhWDR interaction with GhMML4_D12

**Fig. 2.**
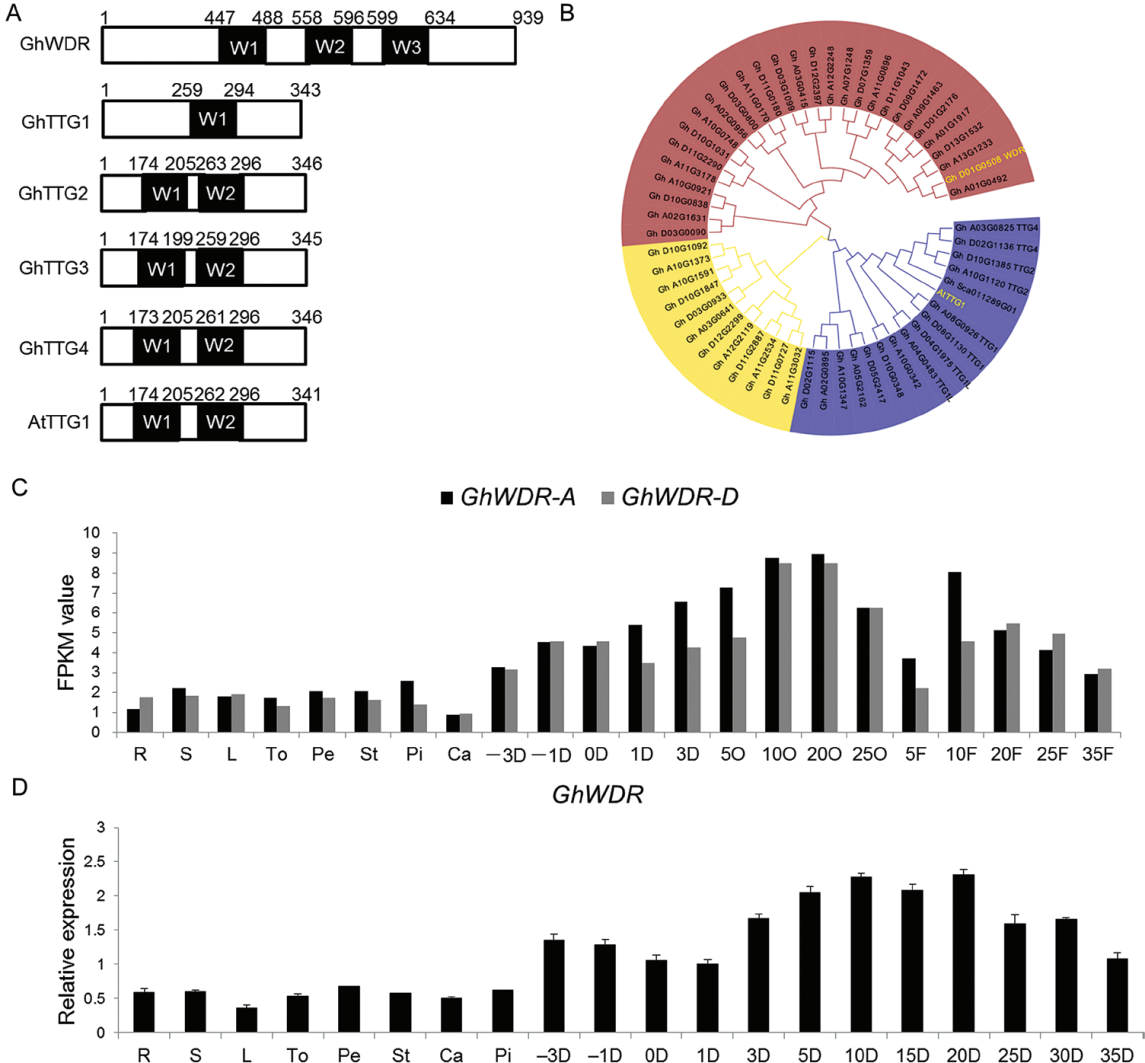
GhWDR belongs to different a clade from GhTTG1 to GhTTG4 and AtTTG1. (A) Schematic diagrams of GhWDR, GhTTG1 to GhTTG4, and AtTTG1 domain constructs. The black box indicates the WD40 repeat domain. Numbers indicate the positions of the first and last amino acids of the domain. (B) GARLI maximum likelihood phylogram of GhWDR, GhTTG1 to GhTTG4 from cotton, and AtTTG1 from Arabidopsis, revealing two different clades. The NJ tree was constructed using the MEGA 6.0 program (http://www.megasoftware.net/). GhWDR belongs to a different clade from GhTTG1 to GhTTG4 and AtTTG1. (C) Fragments per kilobase per million mapped reads (FPKM) value to illustrate the expression pattern of *GhWDR* in various tissues of *G. hirsutum* cv. TM-1. R: root; S: stem; L: leaf; To: torus; Pe: petal; St: stamen; Pi: pistil; Ca: calycle; −3D, −1D, 0D, 1D, and 3D: ovules attached with fibers at −3, −1, 0, 1, and 3 DPA; 5O, 10O, 20O, and 25O: 5, 10, 20, and 25 DPA ovules without fibers; 5F, 10F, 20F, 25F, and 35F: 5, 10, 20, 25, and 35 DPA fibers. (D) qRT-PCR expression analysis of *GhWDR* in various tissues of *G. hirsutum* cv. TM-1. The error bar represents the standard deviation of the mean values of three biological replicates. The *GhUBI1* gene was used as the internal control.

To investigate how *GhMML4_D12* regulates lint fiber development, we used a Y2H assay to identify its potential interaction partners. Full-length GhMML4_D12 CDS was isolated and fused with bait vector (pGBKT7-GhMML4_D12) to screen the cotton fiber specific yeast library. We did not observe any bHLH genes in the Y2H screening, probably due to both *GhMML* genes lacking the core amino acids needed in the interaction with bHLH as reported before (Grotewold *et al.*, 2000; Zimmermann *et al.*, 2004). However, a new kind of WD40 repeat protein (Gh_D01G0508) (Zhang *et al.*, 2015) was found by prototrophy (see Supplementary Table S2). Structural analysis showed that the number of WD40 repeat domains in GhWDR differed from that in AtTTG1 and in GhTTG1 to GhTTG4 (Fig. 2A). Phylogenetic analysis showed that they belong to different clades (Fig. 2B), implying functional differentiation among them. We named this as *GhWDR*.

To better understand this newly found gene, we cloned its genomic DNA and CDS. The *GhWDR* gene contained seven exons and six introns in its open reading frame (ORF), but *GhWDR-A* at the A subgenome had a seven amino acid insertion in its N-terminus (see Supplementary Fig. S1). Both these two genes had three WD40 repeat domains. Both *GhWDR* and *GhTTG1* to *GhTTG4* were expressed in whole ovules at the anthesis day, as well as throughout the elongation and secondary cell wall synthesis stages of fiber development (Fig. 2C, D; Supplementary Fig. S2). qRT-PCR analysis showed that the expression levels of these WD40 genes were lower in fiberless mutant XZ142FLM and n_2_NSM plants compared with the normal TM-1 (Supplementary Fig. S2), suggesting that these genes may contribute to fiber development.

**Fig. 3.**
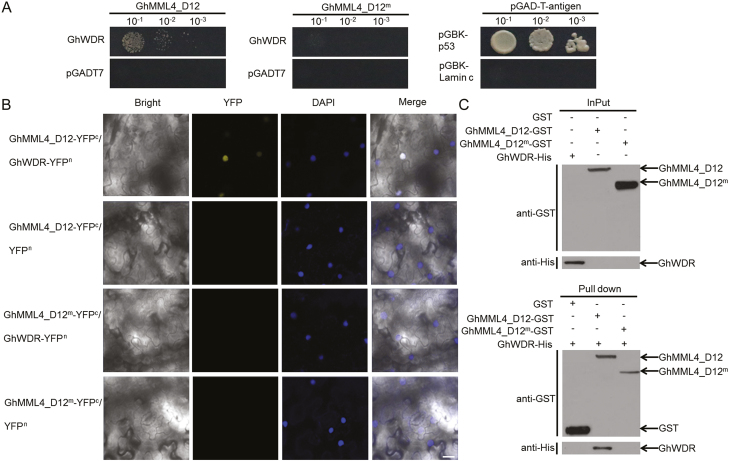
GhWDR could interact with GhMML4_D12, but not with GhMML4_D12^m^. (A) Y2H assays to detect the interactions between GhWDR and GhMML4_D12 or GhMML4_D12^m^. GhMML4_D12 and GhMML4_D12^m^ were fused with pGBKT7, while, GhWDR was fused with pGADT7. Interactions between pGBK-p53 or pGBK-Lamin c and the pGAD-T-antigen were used as positive and negative controls, respectively. (B) BiFC assay to detect the interactions between GhWDR and GhMML4_D12 or GhMML4_D12^m^. GhMML4_D12 and GhMML4_D12^m^ were fused with a cYFP to form cYFP–GhMML4_D12 and cYFP–GhMML4_D12^m^. GhWDR was fused with nYFP to form GhWDR–nYFP. The construct pairs indicated were co-infiltrated into leaves of *N. benthamiana*. YFP fluorescence was detected 50 h after infiltration. The positions of nuclei were indicated by 4′,6-diamidino-2-phenylindole staining. Scale bar: 25 μm. (C) *In vitro* pull down to verify the interaction between GhWDR and GhMML4_D12 or GhMML4_D12^m^. Purified proteins from expression of GST plus His–GhWDR, GST–GhMML4_D12 plus His–GhWDR, and GST–GhMML4_D12^m^ plus His–GhWDR were immunoprecipitated with anti-GST antibody-conjugated agarose and were further detected by immunoblot using an anti-His antibody.

To further confirm the interaction between GhMML4_D12 and GhWDR, full-length GhWDR CDS was introduced into the prey vector (pGADT7-GhWDR), and GhMML4_D12 and GhMML4_D12^m^ introduced into bait vectors (pGBKT7-GhMML4_D12 and pGBKT7-GhMML4_D12^m^, respectively). Bait and prey vectors were co-transformed into yeast and the GhMML4–GhWDR interaction was reconstructed. However, no interaction was observed between GhMML4_D12^m^ and GhWDR (Fig. 3A).

### Validation of interaction between GhWDR and GhMML4_D12

Subcellular localization of GhMML4_D12, GhMML4_D12^m^, and GhWDR revealed that all these proteins were mainly present in the cell nucleus (see Supplementary Fig. S3). Biomolecular fluorescence complementation (BiFC) assays were adopted to validate the interaction between GhWDR and GhMML4_D12 or GhMML4_D12^m^ in leaf epidermal cells in tobacco. The GhWDR protein was fused with the nYFP, and the cYFP was ligated with GhMML4_D12 and GhMML4_D12^m^. GhMML4_D12–cYFP and GhMML4_D12^m^–cYFP were transiently co-expressed with GhWDR–nYFP in tobacco. YFP fluorescence was observed in the nucleus of epidermal cells in tobacco leaves when GhWDR was co-expressed with GhMML4_D12, but no YFP fluorescence was detected when GhWDR was co-expressed with GhMML4_D12^m^ (Fig. 3B).

The interaction between GhWDR and GhMML4_D12 was further confirmed *in vitro*. Purified GST–GhMML4_D12 and GST–GhMML4_D12^m^ fusion proteins were incubated with His–GhWDR expressed in *E. coli*. Bound proteins were washed, separated on SDS-PAGE, and immunoblotted with an anti-GST antibody. As shown in Fig. 3C, the negative control (GST resin) and GST–GhMML4_D12^m^ were unable to pull down His–GhWDR, whereas GST–GhMML4_D12 could efficiently pull down His–GhWDR, suggesting that GhMML4_D12 physically interacts with GhWDR *in vitro*. We have therefore shown for the first time that MML proteins directly interact with WD40 proteins, similar to but different from the MBW complex in regulating Arabidopsis leaf or root hair trichome patterning (Yang and Ye, 2013).

### Decisive domains identified for interaction between GhMML4_D12 and GhWDR

**Fig. 4.**
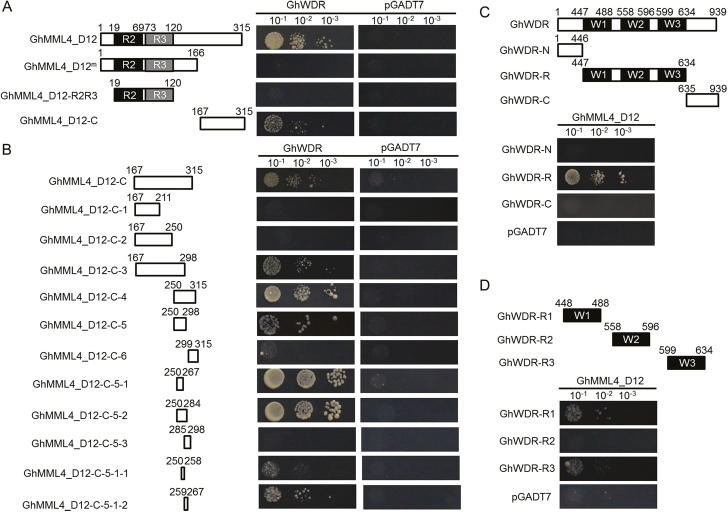
Core domains determining the interaction between GhMML4_D12 and GhWDR. (A) Y2H assays showing that the C-terminus of GhMML4_D12 (GhMML4_D12-C) plays roles in the interaction with GhWDR. Left panel shows schematic diagram of GhMML4_D12, GhMML4_D12^m^, GhMML4_D12-R2R3, and GhMML4_D12-C domain constructs; all of these segments were fused with the DNA binding domain (BD) individually. GhWDR was fused to the activation domain (AD). Protein–protein interactions were assessed on SD/−Trp/−Leu/−His medium with different selective conditions. The grey and black boxes indicate R2 and R3 domains, and the numbers indicate positions of the first and the last amino acids of the domain. (B) Y2H assays showing that amino acids from 250 to 267 of GhMML4_D12 determine the interaction with GhWDR, and amino acids from 259 to 267 are more important. Left panel shows schematic diagram of GhMML4_D12-derived constructs; all these constructs were fused with BD individually. Protein–protein interactions were assessed on SD/−Trp/−Leu/−His medium with different selective conditions. (C) Y2H assays showing that the WD40 repeat domain of GhWDR plays important roles in the interaction with GhMML4_D12. Upper panel shows schematic diagram of GhWDR-N, GhWDR-R, and GhWDR-C; all these domains were fused with AD. Protein–protein interactions were assessed on SD/−Trp/−Leu/−His medium with different selective conditions. (D) Y2H assays showing that the first and third WD40 repeat domains of GhWDR determine the interaction with GhMML4_D12. Upper panel shows schematic diagram of GhWDR-R1, GhWDR-R2, and GhWDR-R3 domain constructs; all these constructs were fused with AD individually. Protein–protein interactions were assessed on SD/−Trp/−Leu/−His medium with different selective conditions.

R2R3 domains and the C-terminus of MYB genes can interact with other transcription factors (Qi *et al.*, 2011, 2014; Song *et al.*, 2011). Since we found that GhMMLs interact with WD40 genes directly, we further investigated how the interaction between GhMML4_D12 and GhWDR occurs. We divided GhMML4_D12 into two parts, GhMML4_D12-R2R3 and GhMML4_D12-C, as shown in Fig. 4A. GhMML4_D12-C was identified as being responsible for the interaction with GhWDR (Fig. 4A). Further investigation showed that amino acids from 250 to 267 of GhMML4_D12 contributed to the interaction with GhWDR, and amino acids from 259 to 267 may be more important (Fig. 4B); however, these amino acids are not conserved in GhMML genes (see Supplementary Fig. S4).

Since many WD40 proteins have been reported to act as transcriptional regulators through protein–protein interactions (Baudry *et al.*, 2004; Zhu *et al.*, 2008), GhWDR was separated into three parts, GhWDR-N, GhWDR-C, and the WD40 repeat domain GhWDR-R. A Y2H assay showed that GhWDR-R was responsible for the interaction with GhMML4_D12, but not GhWDR-N and GhWDR-C (Fig. 4C). GhWDR-R was further divided into three parts based on the number of WD40 repeat domains, namely GhWDR-R1, GhWDR-R2, and GhWDR-R3, as illustrated in Fig. 4D. It was found that GhWDR-R1 and GhWDR-R3 could interact with GhMML4_D12, but the GhWDR-R2 did not (Fig. 4D).

Moreover, we also investigated the interaction between GhMML4_D12 and GhMML4_D12^m^ and other WD40 proteins including GhTTG1 to GhTTG4 and AtTTG1. Interestingly, we found that GhTTG1 to GhTTG4 could interact with both GhMML4_D12 and GhMML4_D12^m^, and there was no obvious difference in the interaction efficacy with these WD40 genes between GhMML4_D12 and GhMML4_D12^m^. However, AtTTG1 was unable to interact with them (see Supplementary Fig. S5), implying a functional divergence of WD40 genes between cotton and Arabidopsis in regulating epidermal cell differentiation. A Y2H assay further showed that the R2R3 domain of GhMML4_D12 was responsible for the interaction with GhTTG1 to GhTTG4, and the C-terminus also participated in the interaction (Supplementary Fig. S5). These results imply that GhWDR is a unique gene that is important for the function of GhMML4_D12; however, the mutated GhMML4_D12^m^ lost its interaction with GhWDR. These results show that amino acids from 250 to 267 of GhMML4_D12 and the first and third WD40 repeat domain of GhWDR are required for the interaction between these proteins.

### GhWDR acts as a transcriptional activator

**Fig. 5.**
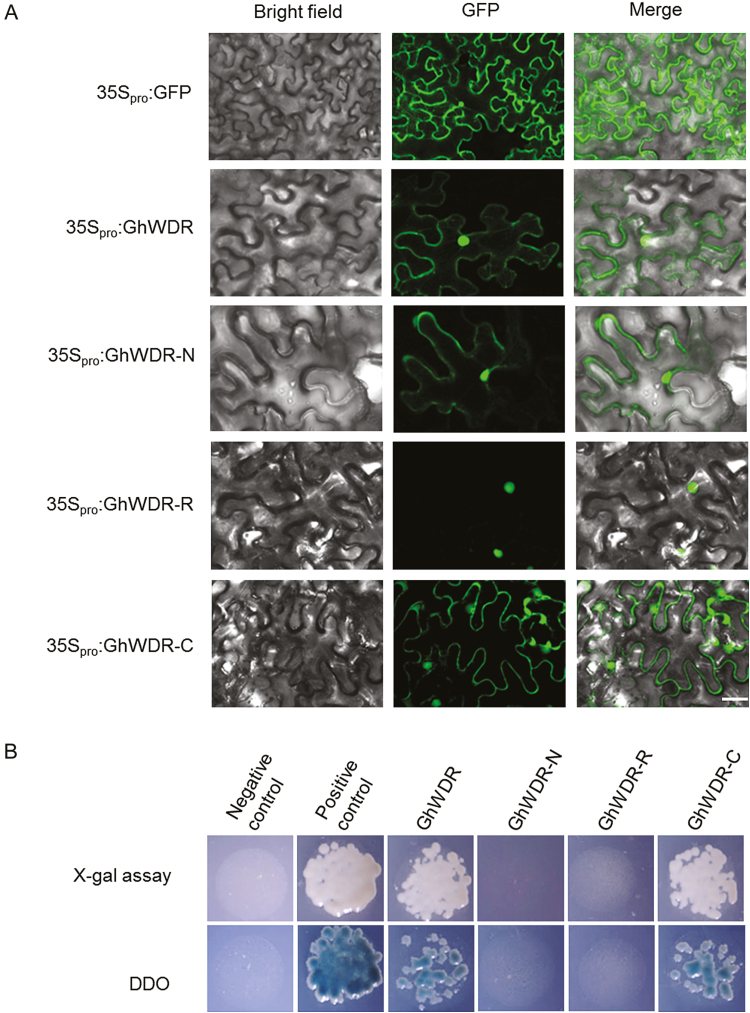
Assay of GhWDR subcellular localization and transcriptional activity. (A) Transient expression of the GhWDR, GhWDR-N, GhWDR-R, and GhWDR-C vectors fused to the N-terminus of GFP (GhWDR–GFP, GhWDR-N–GFP, GhWDR-R–GFP and GhWDR-C–GFP) in epidermal cells of tobacco leaves. Green fluorescence signals representing all the fusion proteins are shown. Scale bar: 25 μm. (B) Transcriptional activity assay of GhWDR and its derivative constructs in yeast. Fusion proteins were expressed in yeast strain AH109. The transformants were streaked on DDO medium (double dropout medium, SD/−Trp/−Ade) and conﬁrmed by ﬂash-freezing ﬁlter assay of the β-galactosidase activity (X-gal assay). pGBKT7-p53 and pGBKT7 were used as the positive and negative controls, respectively.

To investigate the subcellular localization of GhWDR, GhWDR, GhWDR-N, GhWDR-C, and GhWDR-R proteins were each fused in-frame to the 5′ terminus of GFP reporter gene under the control of the CaMV35S promoter and transformed into tobacco leaf cells by *Agrobacterium*, as illustrated in Fig. 4C. The GhWDR and GhWDR-C protein were both detected in the cell nucleus and cytomembrane, GhWDR-R was detected only in the cell nucleus, and GhWDR-N was mainly found in the cell cytomembrane (Fig. 5A).

To identify the transcriptional activity of GhWDR, the yeast GAL4-responsive reporter system were employed. Different domains of GhWDR were fused to the pGBKT7 to generate effector constructs and transform them into yeast. P53-pGBKT7 and pGBKT7 were used as positive and negative controls, respectively. As shown in Fig. 5B, the transformed yeast cells containing GhWDR-pGBKT7 could grow on SD/−Trp to which 50 ng ml^−1^ AbA was added, indicating that the reporter gene, *LacZ*, was activated. Furthermore, the transformed yeast cells containing GhWDR-C-pGBKT7 could grow on SD/−Trp/+100 ng ml^−1^ AbA, whereas those containing GhWDR-N and GhWDR-R were unable to grow on this medium. These results suggest that the GhWDR protein is present in the nucleus, its WD40 domain and C-terminus are sufficient for nuclear localization, and GhWDR functions as a transcriptional activator; the transcriptional activity of GhWDR mainly depends on GhWDR-C.

### Ectopic expression of *GhWDR* rescues *ttg1* mutant phenotypes in Arabidopsis

**Fig. 6.**
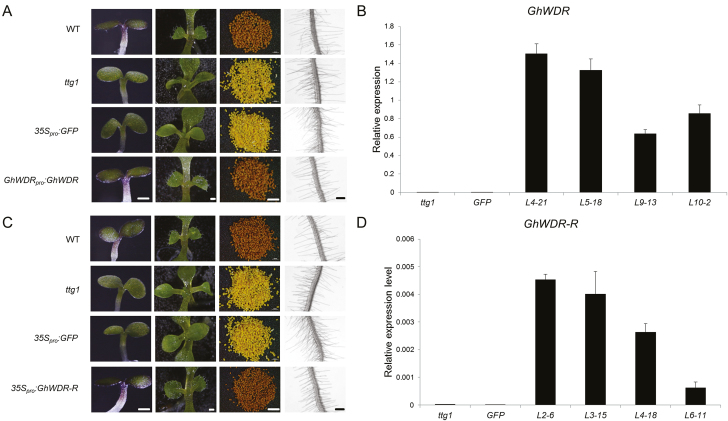
Ectopic expression of *GhWDR* in Arabidopsis *ttg1* rescue anthocyanin accumulation, trichome development, seed coat color, and root hair position. (A) Phenotypic examination of wild type, *ttg1*, *35S*_*pro*_*:GFP*, and *GhWDR*_*pro*_*:GhWDR* plants. First column: anthocyanin accumulation in 10-day-old plants (scale bar: 0.2 mm); second column: trichomes on surfaces of 2-week-old rosette leaves (scale bar: 0.4 mm); third column: mature seeds (scale bar: 0.08 mm); fourth column: root hairs of 5- to 6-day-old seedlings (scale bar: 0.5 mm). (B) qRT-PCR analysis of *GhWDR* expression in 4-week-old rosette leaves from independent Arabidopsis T_2_ lines (L4, L5, L9, and L10), wild types and controls. The *ACT2* gene was used as an internal control. The error bar represents the standard deviation of the mean values of three biological replicates. (C) Phenotypic examination of wild type, *ttg1*, *35S*_*pro*_*:GFP*, and *35S*_*pro*_*:GhWDR-R* plants. Ectopic expression of *GhWDR-R* rescued *ttg1* to its wild type. First column: anthocyanin accumulation in 10-day-old plants (scale bar: 0.2 mm); second column: trichomes on surfaces of 2-week-old rosette leaves (scale bar: 0.4 mm); third column: mature seeds (scale bar: 0.08 mm); fourth column: root hairs of 5- to 6-day-old seedlings (scale bar: 0.5 mm). (D) qRT-PCR analysis of *GhWDR-R* (L1 to L6) expression in 4-week-old rosette leaves from independent Arabidopsis T_2_ lines, wild types and controls. The *ACT2* gene was used as an internal control. The error bar represents the standard deviation of the mean values of three biological replicates.

Arabidopsis trichomes and cotton fibers are both unicellular hairs of the epidermis, and may share a similar molecular machinery of regulation (Wang *et al.*, 2004; Humphries *et al.*, 2005; Guan *et al.*, 2008). Functional investigation of cotton genes in Arabidopsis has been successfully used to elucidate the mechanisms that regulate cotton fiber development. Due to the difficulties of generating transgenic cotton, functional investigation of *GhWDR* was carried out in Arabidopsis glabrous mutant *ttg1*. The *ttg1* allele results in severe defects in anthocyanin synthesis, trichome development, seed coat pigmentation, and root hair position. To investigate whether *GhWDR* could complement *ttg1* mutant phenotypes, we transformed *GhWDR* fused with GFP under the control of a native promoter (*GhWDR*_*pro*_*:GhWDR*) into the *ttg1* mutant. Of the 19 primary kanamycin-resistant T_1_ transgenic lines, four (L4, L5, L9, and L10) with ectopic expression of *GhWDR* showed the wild type phenotype, whereas no trichomes were observed in non-transformed (indicating as *ttg1*) and empty-vector controls (indicating as *35S*_*pro*_*:GFP*) (Fig. 6A). qRT-PCR analysis showed higher levels of ectopic expression of *GhWDR* in these four independent T_2_ lines, which could rescue *ttg1* to its wild type phenotype (Fig. 6B).

The WD40 repeat domain of GhWDR decided the interaction with GhMML4_D12, and we were interested in whether it could complement *ttg1* mutant phenotypes. We transformed *GhWDR-R* fused to *GFP* driven by CaMV35S (*35S*_*pro*_*:GhWDR-R*) into *ttg1* Arabidopsis mutants. Phenotype examination revealed that no T_1_*GhWDR-R* overexpression plants had wild type anthocyanin synthesis or trichome development. After all seeds were harvested, we were surprised to find that some of the mature seeds from *GhWDR-R* overexpression T_1_ lines were brown, similar to the wild type (Fig. 6C), indicating that *GhWDR-R* could rescue *ttg1* to its wild type. Higher expression levels of *GhWDR-R* were examined in all those T_2_ lines that also rescue *ttg1* to wild type (Fig. 6D).

Together, these results reveal that, similar to Arabidopsis *TTG1*, *GhWDR* plays an important orthologous role in anthocyanin synthesis, trichome development, seed coat pigmentation and root hair position, confirming its function in trichome development. Further investigation showed that its WD40 repeat domain is important for the function of *GhWDR* in *ttg1* Arabidopsis.

### GhMML4_D12 promotes transcriptional activation of itself in n_2_NSM

**Fig. 7.**
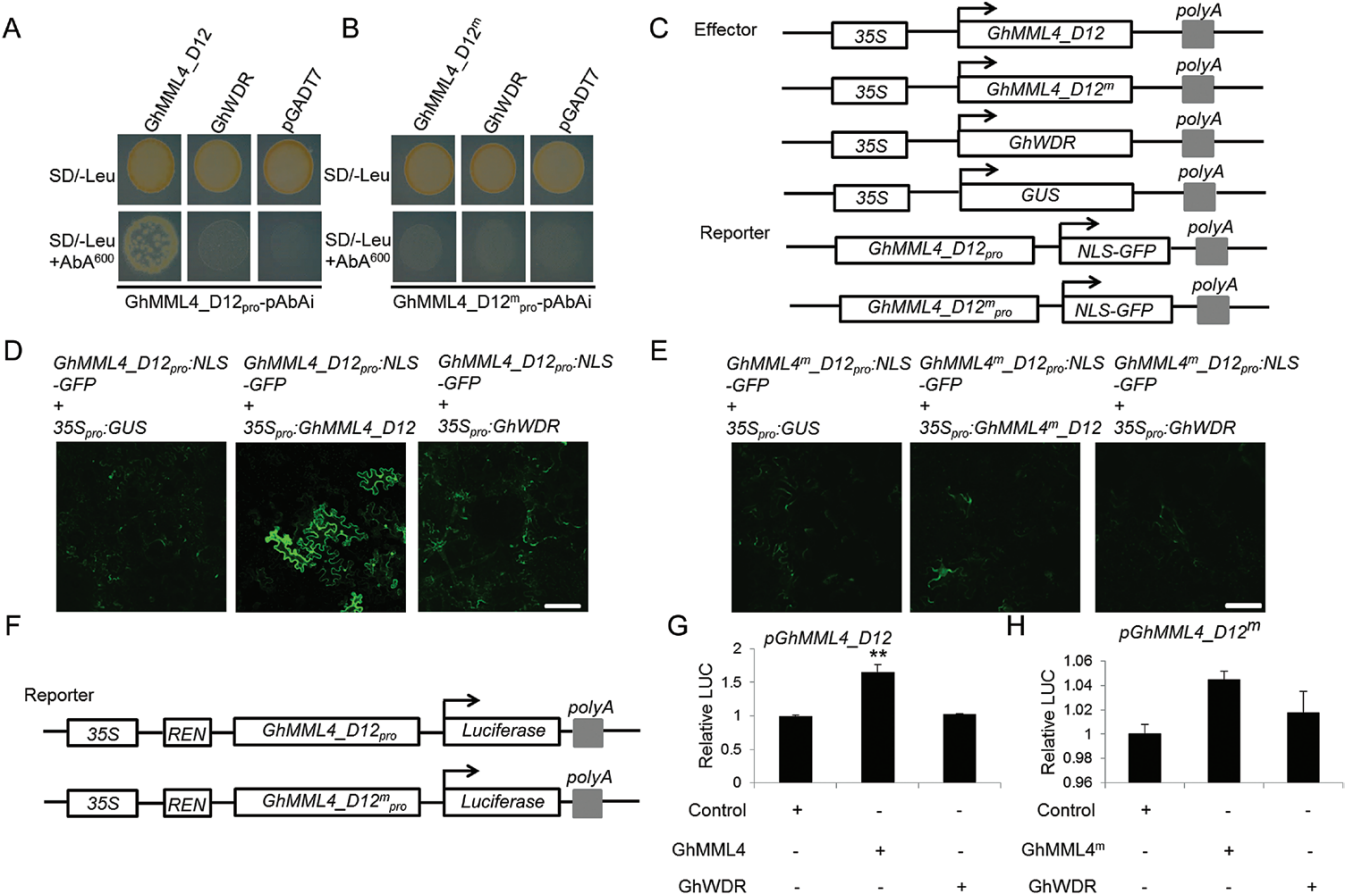
GhMML4_D12 enhance the transcriptional activity of itself in n_2_NSM, but GhMML4_D12^m^ did not in XZ142FLM. (A, B) Y1H analysis show that GhMML4_D12 binds to its own promoter in n_2_NSM, but GhMML4_D12^m^ did not in XZ142FLM. AbA, a yeast cell growth inhibitor, was used as a screening marker. The basal concentration of AbA was 600 ng ml^−1^. Co-transformed empty vectors with the *GhMML4_D12* and *GhMML4_D12*^*m*^ promoters were used as negative controls. (C) Schematic diagram of *GhMML4_D12*_*pro*_*:NLS-GFP* and *GhMML4_D12*^*m*^_*pro*_*:NLS-GFP* reporters and *35S*_*pro*_*:GhMML4_D12*, *35S*_*pro*_*:GhMML4_D12*^*m*^, *35S*_*pro*_*:GhWDR*, and *35S*_*pro*_*:GUS* effectors. (D, E) Transient expression assays show that GhMML4_D12 activates the expression of *GhMML4_D12*_*pro*_*:NLS-GFP* in n_2_NSM, but GhMML4_D12^m^ did not activate the expression of *GhMML4_D12*^*m*^_*pro*_*:NLS-GFP* in XZ142FLM. GhWDR did not activate the expression of either *GhMML4_D12*_*pro*_*:NLS-GFP* in n_2_NSM or *GhMML4_D12*^*m*^_*pro*_*:NLS-GFP* in XZ142FLM. GFP fluorescence was detected 48 h after co-infiltration with the indicated constructs. The experiment was repeated three times with similar results and representative photos are displayed. Scale bar: 50 μm. (F) Schematic representation of the reporters used in the transient Dual-LUC assay; effectors were the same as in (C). (G, H) Transient Dual-LUC reporter assays show that GhMML4_D12 enhances its own transcriptional activity in n_2_NSM, but GhMML4_D12^m^ did not in XZ142FLM. GhWDR did not affect the transcriptional activity of either *GhMML4_D12* in n_2_NSM or *GhMML4_D12*^*m*^ in XZ142FLM. Three independent transfection experiments were performed. The error bar represents the standard deviation of the mean values of three biological replicates. Asterisks indicate significantly different values (**P<0.01).

An increasing number of studies have shown that transcription factors can bind to the promoter of their target genes to either activate or repress expression (Chen *et al.*, 2017; Lei *et al.*, 2017; Zhang *et al.*, 2018). To understand the relationship between *GhMML4_D12* and *GhWDR*, we employed Y1H analysis to investigate the effect of GhMML4_D12, GhMML4_D12^m^, and GhWDR on the transcriptional activity of *GhMML4_D12*. GhMML4_D12, GhMML4_D12^m^, and GhWDR were fused with pGADT7, and the promoters of *GhMML4_D12* from n_2_NSM and XZ142FLM (*GhMML4_D12*^*m*^) were ligated into pAbAi. Sequence comparison of these two promoters showed no notable difference between them (see Supplementary Fig. S6). As shown in Fig. 7A, B, GhMML4_D12 could bind to its own promoter in n_2_NSM, but GhMML4_D12^m^ could not in XZ142FLM. To further verify the results, *35S*_*pro*_*:GhMML4_D12*, *35S*_*pro*_*:GhMML4_D12*^*m*^, *35S*_*pro*_*:GhWDR*, and *35S*_*pro*_*:GUS* were used as effector plasmids. *NLS* was ligated at the 3′ end of *GhMML4_D12* and *GhMML4_D12*^*m*^ promoters, then fused with GFP at the 3′ end of *NLS* to construct *GhMML4_D12*_*pro*_*:NLS-GFP* and *GhMML4_D12*^*m*^_*pro*_*:NLS-GFP*. These two constructs were used as reporter plasmids (Fig. 7C). Co-transformation of these two reporters with *35S*_*pro*_*:GUS* into tobacco leaves resulted in a relatively low level of fluorescence signal (Fig. 7D, E). However, when *35S*_*pro*_*:GhMML4_D12* was infiltrated into tobacco leaves, a much stronger fluorescence signal was observed (Fig. 7D). In contrast, co-infiltration of *GhMML4_D12*_*pro*_*:NLS-GFP* with *35S*_*pro*_*:GhWDR* generated relatively low levels of fluorescence (Fig. 7D). However, co-infiltration of *GhMML4_D12*^*m*^_*pro*_*:NLS-GFP* with either *35S*_*pro*_*:GhMML4_D12*^*m*^ or *35S*_*pro*_*:GhWDR* had no effect (Fig. 7E).

To confirm these results, we further employed a dual-luciferase (Dual-LUC) reporter approach. *35S*_*pro*_*:GhMML4_D12*, *35S*_*pro*_*:GhMML4_D12*^*m*^, *35S*_*pro*_*:GhWDR*, and *35S*_*pro*_*:GUS* were used as effector plasmids, In addition, the reporters consisted of 35S promoter-driven *Renilla* luciferase (*REN*, as internal control) and *GhMML4_D12* or *GhMML4_D12*^*m*^ promoter-driven firefly luciferase (Fig. 7F). The expression of *35S*:*GhMML4_D12* increased the LUC/REN ratio (Fig. 7G), while co-expression *35S*_*pro*_*:GhWDR* had little influence on *GhMML4_D12* activity compared with *35S*_*pro*_*:GUS* alone (Fig. 7G). Co-expression of *35S*_*pro*_*:GhMML4_D12*^*m*^ and *35S*_*pro*_*:GhWDR* had no effect on *GhMML4_D12*^*m*^ activity compared with *35S*_*pro*_*:GUS* alone (Fig. 7H). All these results suggest that GhMML4_D12 enhances the transcriptional activity of itself in n_2_NSM, but not in XZ142FLM, and the transcriptional activity may improve the function of GhMML4_D12 in regulating cotton lint fiber development.

The transcriptional regulation of GhMML4_D12, GhMML4_D12^m^ and GhWDR on *GhWDR* was also investigated. We found that neither GhMML4_D12 nor GhMML4_D12^m^ was able to activate the expression of *GhWDR*, and GhWDR was also unable to activate its own transcriptional activity (see Supplementary Fig. S7), implying GhWDR simply serves as a scaffolding molecule in the protein-protein interactions to assist the function of GhMML4_D12.

## Discussion

### Evolution of trichome differentiation-related genes in plants

The MYB gene family members, which largely function as transcription factors, have several conserved binding domains. Of these, R2R3 MYB is the largest subgroup. Subgroup 9 of the R2R3 MYBs contains *MML* genes and subgroup 15 contains *GL1-Like* genes. These genes play important roles in specification and regulation of plant cellular differentiation (Paterson *et al.*, 2012; Brockington *et al.*, 2013). *MML* genes mainly control fiber development. The *GL1-Like* gene *GaMYB2* complements Arabidopsis *gl1*, and its ectopic expression induces a single trichome from the epidermis of Arabidopsis seeds (Wang *et al.*, 2004). Although *GaMYB2* is highly expressed in developing fibers, some other genes, *GhMYB25*, *GhMML3_A12*, and *GhMML4_D12*, belong to a novel *MIXTA* clade of MYB transcription factors involved in cotton seed fiber differentiation (Machado *et al.*, 2009; Walford *et al*, 2011; Wan *et al.*, 2016; Wu *et al.*, 2018). Expression of these *MML* genes is restricted to cotton ovule epidermal cells and young fiber cells, not trichomes on other plant parts, such as stems, leaves, and petals (Zhang *et al.*, 2015). These *MML* genes are more distinct in cotton fibers and have independent ovule-specific functions that directly expand epidermal cells for seed trichome or fiber production. These genes have closer phylogenetic relationships with *MML* genes expressed in petals than some other MYB genes (*GL1* clade) associated with other types of trichome or root hairs. So, these *MML* transcription factors have likely evolved independent ovule-specific functions to direct the expansion of epidermal cells to produce the cotton fiber. These findings will also be helpful to illustrate the roles of *MIXTA* genes in regulating epidermal cell differentiation such as production of a ‘pulpy layer’ secreted from the teguments surrounding cacao seeds, and mucilages in other Malvaceae fruit (*Abelmoschus* (okra), and *Cola* (kola)).

WD40 repeat proteins are characterized by the presence of 40–60 amino acid peptide motifs, which are usually delimited by the GH dipeptide (Gly-His) and the WD dipeptide (Trp-Asp) at the C- and N-termini (van Nocker and Ludwig, 2003). WD40 proteins are found widely in the plant kingdom and regulate the biosynthesis of anthocyanin, proanthocyanin, and mucilage in seeds, and the development of trichomes and root hairs. The *TTG1* locus regulates several developmental and biochemical pathways in Arabidopsis, including the formation of hairs on leaves, stems, and roots, as well as the production of seed mucilage and anthocyanin pigments (Walker *et al.*, 1999). Two cotton WD40 transcription factors, *GhTTG1* and *GhTTG3*, could rescue *ttg1* to its wild type (Humphries *et al.*, 2005). Phylogenetic analysis showed that *GhWDR* belongs to a clade that is different from that of *AtTTG1* and *GhTTG1* to *GhTTG4*, implying functional divergence between them (Fig. 2B). *GhWDR* could complement *ttg1* to its wild type, implying its conservative function in trichome development (Fig. 6A). Further investigation showed that the WD40 repeat domain (from amino acid 454 to 773) of *GhWDR* could rescue *ttg1* (Fig. 6C). Further transgenic analysis of *GhWDR* in cotton fiber development is needed in future work to help in understanding the key role of the WD40 family in plants.

### GhMML4–GhWDR complex shows similarity to but is distinct from MBW complex

The MBW ternary complex consists of MYB, bHLH, and WD40 transcription factors, and has important roles in anthocyanin and proanthocyanin biosynthesis and trichome initiation in a variety of plant species. Usually, WD40 proteins do not interact with MYB proteins directly; they just serve as scaffolding molecules to interact with bHLH factors, and then bHLH factors interact with different kinds of MYB factors to regulate different cell fates (Ramsay and Glover, 2005). The MYB–bHLH complex regulates distant and diverse biological processes such as cell death, cell wall synthesis, hormone signaling, stamen development, and seed production (Stracke *et al.*, 2001). Further investigation of protein–protein interaction specificities of the MYB protein in Arabidopsis revealed a conserved amino acid signature ([DE]Lx2[RK]x3Lx6Lx3R) as the structural basis for the interaction between MYB and R/B-like bHLH proteins (Zimmermann *et al.*, 2004).

In this study, we found a lint fiber-specific *MML* factor, GhMML4_D12, could directly interact with a newly found WD40 factor, GhWDR, but not with any bHLH genes. And no interaction between GhMML4_D12^m^ and GhWDR was observed (Fig. 3). The newly found GhMML4–GhWDR complex shares some similarity to but is distinct from the MBW ternary complex in regulation of leaf/root hair trichome patterning in Arabidopsis. The similarity between them is that the members of these two complexes both belong to the MYB, bHLH, or WD40 factors, and thus GhMML4_D12 and GhWDR have diverged from R2R3MYB and WD40. Meanwhile, the combining form is different.

We believe cotton fiber (seed trichome) and leaf/root hair trichomes have a similar but different regulatory network. Both Arabidopsis trichomes and cotton fibers are unicellular structures of epidermal origin. And they may share similar molecular machinery of regulation. Several genes implicated in cotton fiber development were shown to functionally substitute for their homologues in Arabidopsis. Ectopic expression of *GaMYB2* complements *gl1* mutant in Arabidopsis (Wang *et al.*, 2004). Moreover, two cotton WD40 transcription factors, *GhTTG1* and *GhTTG3*, are able to rescue Arabidopsis *ttg1* to its wild type (Humphries *et al.*, 2005). Furthermore, one *HOX* gene, *GaHOX1*, is a functional homologue of Arabidopsis *GL2* (Guan *et al.*, 2008). Therefore, it had been supposed for a long time that development of cotton fibers and Arabidopsis trichomes shares a similar mechanism. However, Machado *et al*. (2009), Walford *et al*. (2011), and our previous reports (Wan *et al.*, 2016; Wu *et al.*, 2018) all found that *GhMML3_A12* and *GhMML4_D12*, belong to a novel *MIXTA* clade of MYB transcription factors, involve in cotton fiber differentiation. So, the genes controlling Arabidopsis trichome and cotton fiber development may be different, although unbranched cotton ﬁbers and trichomes share some degree of morphological similarity. For example, Arabidopsis *MYBMIXTA-Like* (*MML*) gene *AtMYB106* was a negative regulator of branching in Arabidopsis, while its homologue *GhMYB25* is reported to be a key regulator for cotton ﬁber initiation (Machado *et al.*, 2009; Oshima *et al.*, 2013). Furthermore, spontaneous fiberless mutants showed normal trichome development (Ruan *et al.*, 2000; Du *et al.*, 2001; Ruan, 2005), and genes regulating fiber initiation and leaf trichomes have been mapped to different loci (Loguerco *etal*., 1999). All this evidence confirms that cotton fibers and leaf trichomes may be regulated by different mechanisms.

Due to the similarity and difference between the GhMML4–GhWDR complex and MBW complex, therefore, we report here that cotton fibers and Arabidopsis leaf/hair trichomes may have similar but different regulatory networks in plants. Further investigation is needed to uncover the mechanisms underlying cotton fiber development and trichome formation in other organs of the plant.

**Fig. 8.**
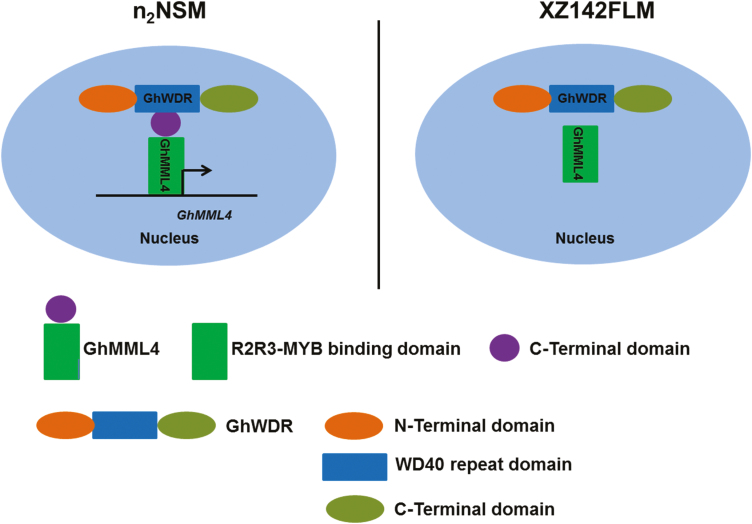
A proposed model of the mechanism by which the GhMML4–GhWDR regulatory complex regulates cotton lint fiber development. In n_2_NSM, the C-terminal domain of GhMML4_D12 directly interacts with the WD40 repeat domain of GhWDR; GhMML4_D12 bound to its own promoter to enhance its transcriptional activity in regulation of lint fiber development. However, in XZ142FLM, mutated GhMML4_D12^m^ did not interact with GhWDR, and thus GhMML4_D12^m^ could not activate its own transcriptional activity. The green box indicates R2R3 domains; the purple circle indicates C-terminal domain of GhMML4_D12; orange and light green circles indicates N-terminal domain and C-terminal domain of GhWDR, respectively; and the blue box indicates WD40 repeat domain of GhWDR.

In summary, the C-terminal domain of GhMML4_D12 and WD40 repeat domain of GhWDR determine their interaction in n_2_NSM. GhWDR simply serves as a scaffolding molecule in protein–protein interactions to assist the function of GhMML4_D12. However, the mutated GhMML4_D12^m^ has lost the C-terminal domain, and therefore could not interact with GhWDR in XZ142FLM. GhMML4_D12 bound to its own promoter to enhance its transcriptional activity, and this may help in the function of GhMML4_D12 in n_2_NSM, but GhMML4_D12^m^ did not do this in XZ142FLM (Fig. 8). These together provide a new insight into the function of GhMML4_D12 in the regulation of cotton lint fiber development in n_2_NSM.

## Supplementary data

Supplementary data are available at *JXB* online.

Fig. S1. Cloning and characterization of *GhWDR*.

Fig. S2. Expression pattern analysis of WD40 genes in fiber mutants.

Fig. S3. Subcellular localization of GhMML4_D12, GhMML4_D12^m^, and GhWDR protein in leaf cells of tobacco.

Fig. S4. Alignment of amino acids from 10 *GhMMLs* genes in cotton.

Fig. S5. Interaction between GhMML4_D12, GhMML4_D12^m^ and GhTTG1–GhTTG4, AtTTG1 in yeast.

Fig. S6. Sequence comparison of *GhMML4_D12* promoters from TM-1, n_2_NSM, and XZ142FLM.

Table S1. All primers developed and used in present research.

Table S2. Proteins interacting with GhMML4_D12 identified by Y2H screening.

eraa104_suppl_supplementary_figures_S1-S7_tables_S1-S2

## Abbreviations

AbA: aureobasidin A
BiFC: biomolecular fluorescence complementation
CDS: coding sequence
DPA: days post-anthesis
Dual-LUC: dual-luciferase
NLS: nuclear localization site
ORF: open reading frame
qRT-PCR: quantitative reverse transcription polymerase chain reaction
REN: *Renilla* luciferase
YFP: yellow fluorescent protein
Y1H: yeast one-hybrid
Y2H: yeast two-hybrid.

## Accession numbers

Sequence data for the genes described in this study can be found in the GenBank database under the following accession numbers: *AtTTG1* (NP_001318637), *GhTTG1* (AAM95642.1), *GhTTG2* (NP_001313775), *GhTTG3* (XP_012460128), *GhTTG4* (XP_016693970) and *GhWDR* (XP_016702485).
