# Supplementary material for: The transcription factor MML4_D12 regulates fiber development through interplay with the WD40-repeat protein WDR in cotton
Source: J Exp Bot. 2020 Mar 2;71(12):3499–511. doi: 10.1093/jxb/eraa104 (PMC7475258; doi:10.1093/jxb/eraa104)
Supplement: eraa104_suppl_supplementary_figures_S1-S7_tables_S1-S2 [file eraa104_suppl_supplementary_figures_s1-s7_tables_s1-s2.pdf]

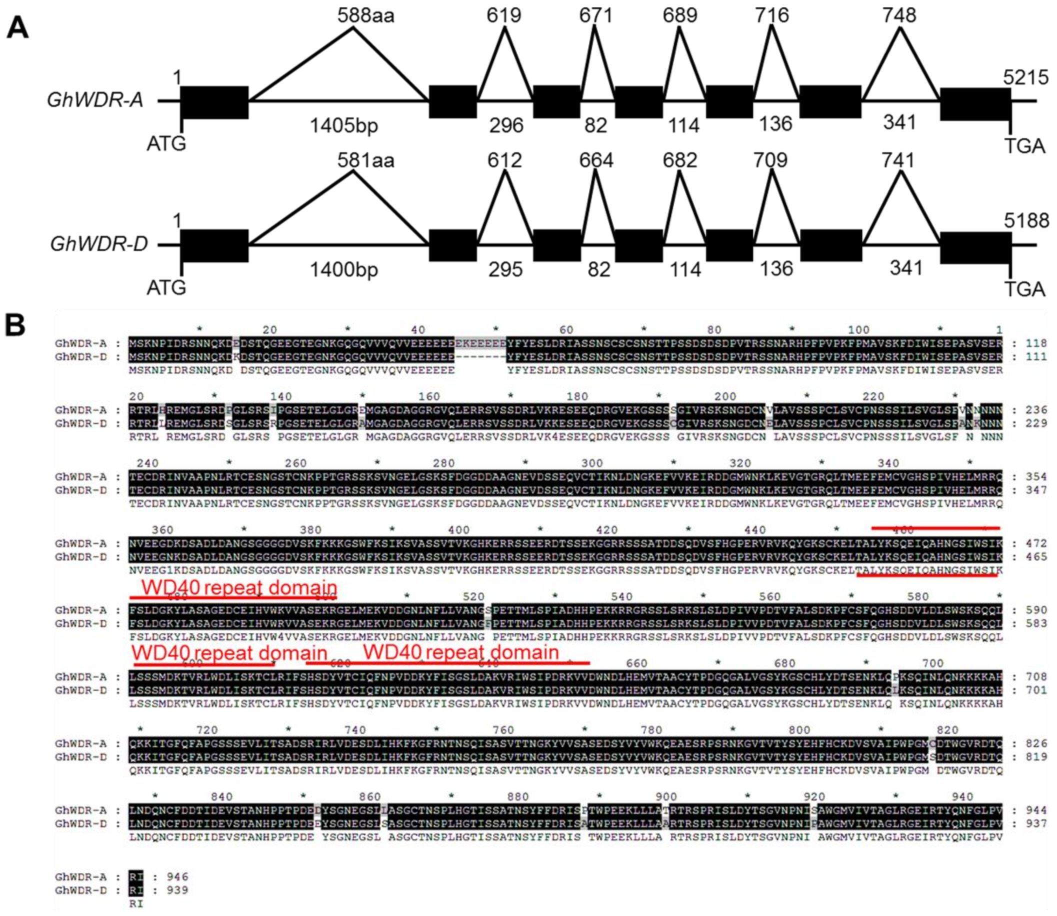

Fig. S1 Cloning and characterization of *GhWDR*.

(A) Schematic digram of cotton (*Gossypium hirsutum*) *GhWDR* gene. The length of both copies of *GhWDR* coding region is around 5.2 kb, containing 7 exons and 6 introns. (B) The homoeologous GhWDR-A and GhWDR-D are 98% identical with amino acid sequences. Proteins belong to WD40 family. Conserved domains of WD40 proteins are underlined in red.

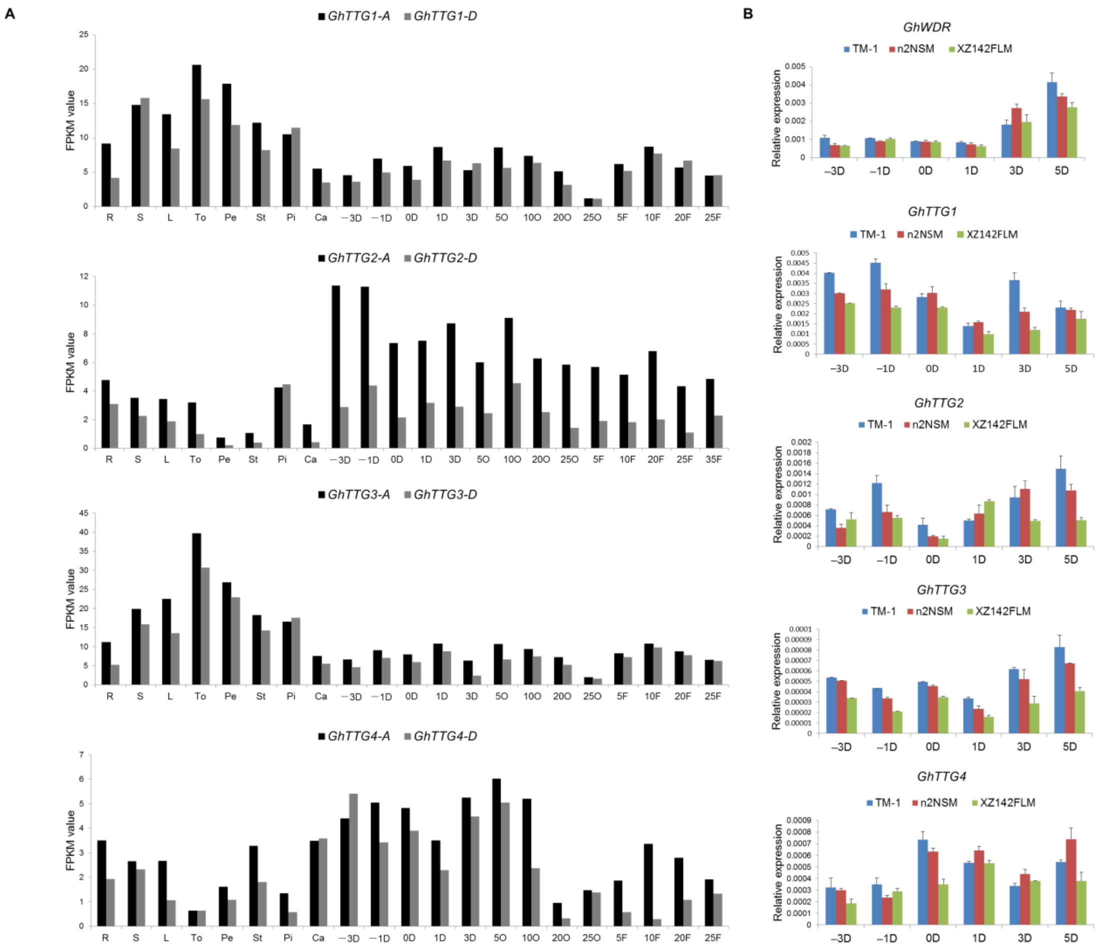

Fig. S2 Expression pattern analysis of WD40 genes in fiber mutants.

(A) FPKM value to illustrate the expression pattern of WD40 genes in various tissues of *G.hirsutum* cv. TM-1. R: root; S: stem; L: leaf; To: torus; Pe: petal; St: stamen; Pi: pistil; Ca: calycle; −3D, −1D, 0D, 1D, 3D: ovules attached with fibers at −3, −1, 0, 1 and 3 DPA; 5O, 10O, 20O and 25O: 5, 10, 20 and 25 DPA ovules without fibers; 5F, 10F, 20F, 25F and 35F: 5, 10, 20, 25 and 35 DPA fibers. (B) qRT-PCR analysis of WD40 genes in TM-1, n_2NSM and XZ142FLM. −3, −1, 0, 1, 3 and 5 D: −3, −1, 0, 1, 3 and 5 DPA ovules attached with fibers.

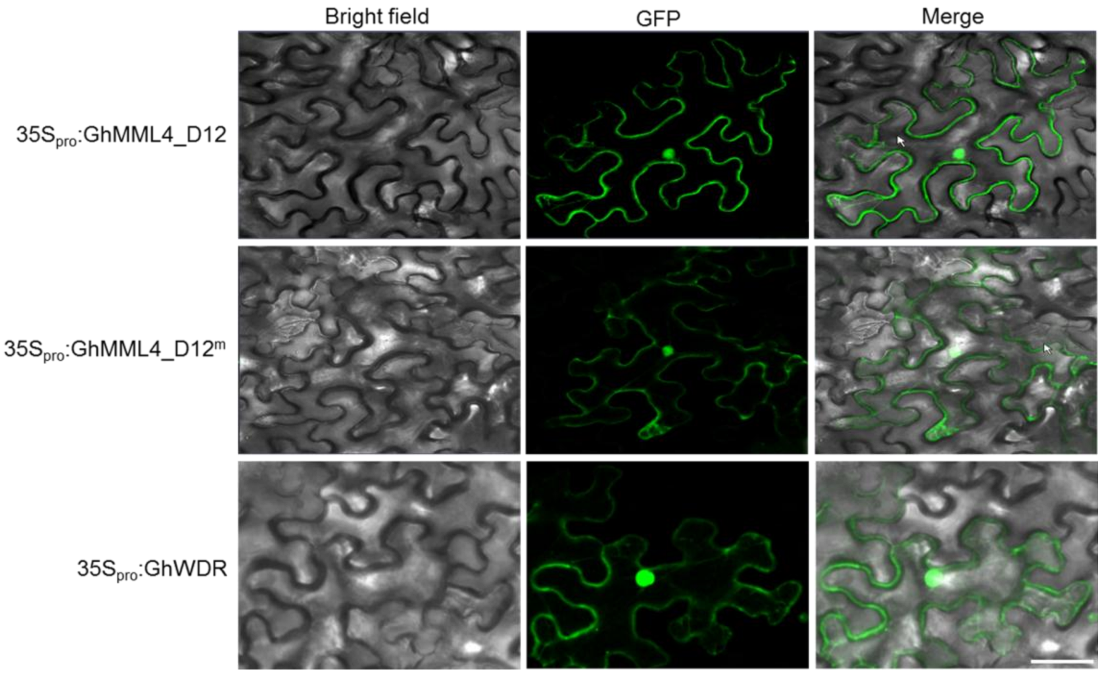

Fig. S3 Subcellular localization of GhMML4_D12, GhMML4_D12^m and GhWDR protein in leaf cells of tobacoo.

GFP fluorescence proteins were fused to the GhMML4_D12, GhMML4_D12^m and GhWDR respectively, and transiently expressed in *N. benthamiana* leave cells via *Agrobacteria* infiltration. All the fluorescence signals were localized to the nucleus. Scale bars = 20 μm.

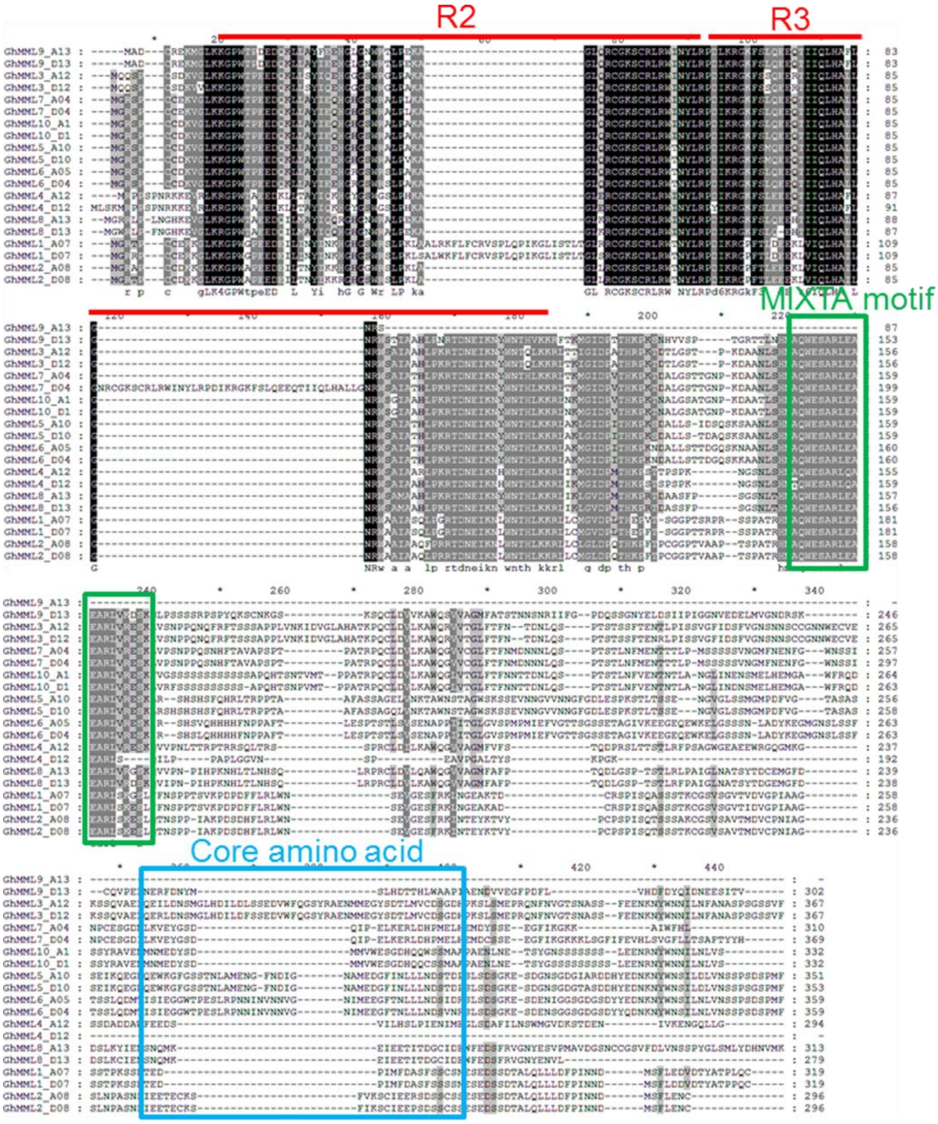

Fig. S4 Alignment of amino acid from ten *GhMMLs* genes in cotton.

Red lines represent R2R3 domain of MYB gene family, green box represents MIXTA motif of *MMLs* genes, blue box represents core amino acids of GhMML4_D12 that interact with GhWDR.

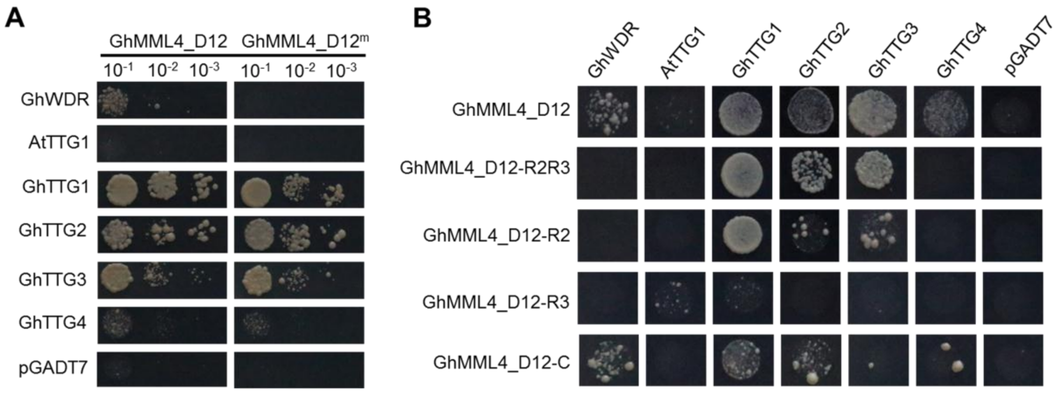

Fig. S5 Interaction between GhMML4_D12, GhMML4_D12^{m} with GhTTG1 to GhTTG4, AtTTG1 in yeast.

(A) Interaction between full length of GhMML4_D12, GhMML4_D12^{m} and GhTTG1 to GhTTG4, AtTTG1. GhMML4_D12, GhMML4_D12^{m} were fused with DNA binding domain (BD), GhTTG1 to GhTTG4, AtTTG1 were fused with activation domain (AD). Protein–protein interactions were assessed on SD/-Trp/-Leu/-His with different selective conditions. (B) GhMML4_D12-R2R3, GhMML4_D12-R2, GhMML4_D12-R3, GhMML4_D12-C were fused with BD. Interactions between GhMML4_D12-R2R3, GhMML4_D12-R2, GhMML4_D12-R3, GhMML4_D12-C with GhTTG1 to GhTTG4, AtTTG1 were assessed on SD/-Trp/-Leu/-His with different selective conditions.

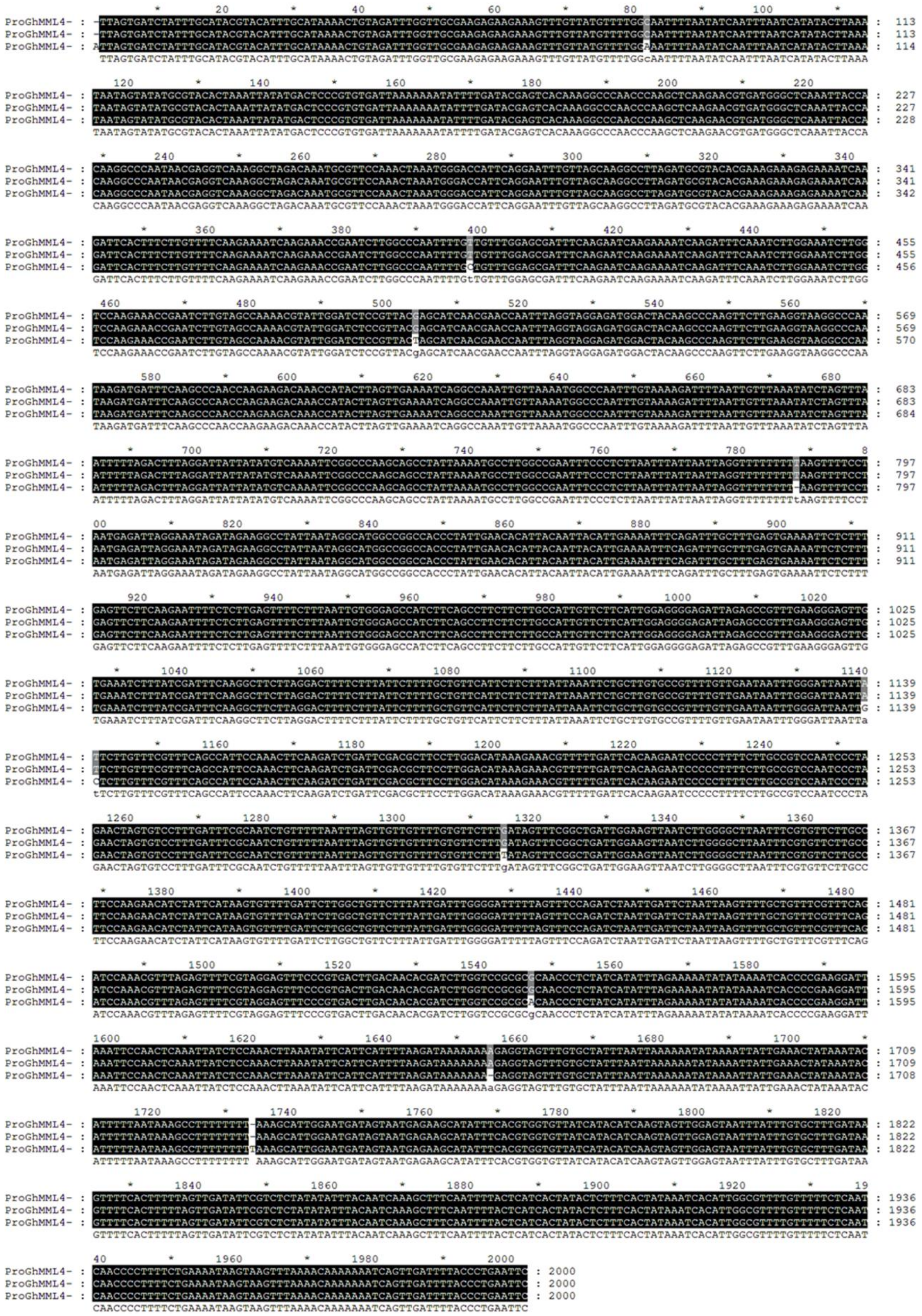

Fig. S6 Sequence comparison of *GhMML4_D12* promoters from TM-1, n_2NSM and XZ142FLM.

2000 bp sequence upstream of *GhMML4_D12* ATG from TM-1, n_2NSM and XZ142FLM were cloned and compared, the first line was from TM-1, the second line

was from n_2NSM, and the third line was from XZ142FLM. Promoters of *GhMML4_D12* from TM-1 and n_2NSM were identical with each other, and promoter of *GhMML4_D12*^{m} from XZ142FLM was slightly different with them.

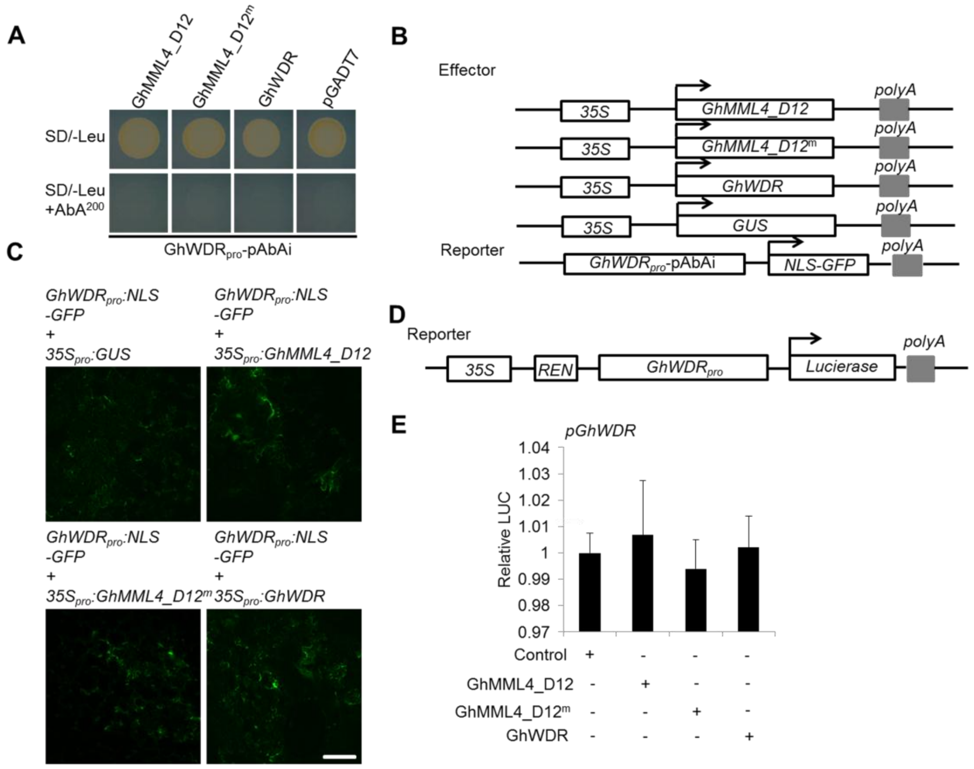

Fig. S7 *GhMML4_D12* and *GhMML4_D12* ^{m} could not bind to the promoter of *GhWDR*.

(A) Y1H assays show that both *GhMML4_D12* and *GhMML4_D12* ^{m} could not bind to the promoter of *GhWDR*. AbA (Aureobasidin A), a yeast cell growth inhibitor, was used as a screening marker. The basal concentration of AbA was 200 ng ml^{-1}. The empty vector and the *GhWDR* promoter was used as negative control. (B) Schematic diagram of the *GhWDR*$_{pro}$*:NLS-GFP* reporter and *GhMML4_D12*, *GhMML4_D12* ^{m}, *GhWDR* and *GUS* effectors. (C) Transient expression assays show that both *GhMML4_D12* and *GhMML4_D12* ^{m} could not affect the transcriptional activity of *GhWDR*$_{pro}$*:NLS-GFP*, fluorescence was detected 48 h after co-infiltration with the indicated constructs. The experiment was repeated three times with similar results and representative photos were displayed. Scale bar = 50 μm. (D) Schematic of the reporters and effectors used in the Dual-LUC assays. (E) Transient Dual-LUC reporter assays show that both *GhMML4_D12* and *GhMML4_D12* ^{m} could not activate the expression of *GhWDR*.

Table S1 All primers developmented and used in present research.

Primer name	Forward primer 5'-3'	Reverse primer 5'-3'	Purpose
K3086	TCCCCCGGGGCCATGCTGAGCAAAATGCGGCCA	TTCCAATGCATTGGCTGCAGGTTATCCCAACACAGGCGAACCA	Yeast two hybrid
K3087	TCCCCCGGGGCCATGCTGAGCAAAATGCGGCCA	TTCCAATGCATTGGCTGCAGGTTACTCACGGACCAACCTGGC	Yeast two hybrid
K4311	CCGGAATTCATGCTGAGCAAAATGCGGCCA	CGCGGATCCTTTCTTTAGATGTGTGTTCCA	Yeast two hybrid
K4312	CCGGAATTCATGCTGAGCAAAATGCGGCCA	CGCGGATCCAGGTCTTAAGTAGTTAATCCA	Yeast two hybrid
K4313	CCGGAATTCAGAGGAAAGTTTAGTTTA	CGCGGATCCTTTCTTTAGATGTGTG	Yeast two hybrid
K4314	CCGGAATTCAAACAGGTTGTCCCAAAT	CGCGGATCCTTATCCCAACACAGGC	Yeast two hybrid
K7775	TCAGAGGAGGACCTGCATATGAAACAGGTTGTCCCAAAT	TCGACGGATCCCCGGGAATTCCTGGGTGGAGAAAACAAAC	Yeast two hybrid
K7776	TCAGAGGAGGACCTGCATATGAAACAGGTTGTCCCAAAT	TCGACGGATCCCCGGGAATTCTGAGTCCTCCTCAAACCA	Yeast two hybrid
K7777	TCAGAGGAGGACCTGCATATGAAACAGGTTGTCCCAAAT	TCGACGGATCCCCGGGAATTCATCCCAGCAATTACCA	Yeast two hybrid
K7821	TCAGAGGAGGACCTGCATATGGAGGAGGACTCAGTCA	TCGACGGATCCCCGGGAATTCTTATCCCAACACAGGC	Yeast two hybrid
K7822	TCAGAGGAGGACCTGCATATGGAGGAGGACTCAGTCA	TCGACGGATCCCCGGGAATTCATCCCAGCAATTACCA	Yeast two hybrid
K7823	TCAGAGGAGGACCTGCATATGAGCGTACTCAACTTC	TCGACGGATCCCCGGGAATTCTTATCCCAACACAGG	Yeast two hybrid
K7824	TCAGAGGAGGACCTGCATATGGAGGAGGACTCAGTCA	TCGACGGATCCCCGGGAATTCTTCCATTATATTTGC	Yeast two hybrid
K7825	TCAGAGGAGGACCTGCATATGGAGGAGGACTCAGTCA	TCGACGGATCCCCGGGAATTCTGATTTGTCGACACCCA	Yeast two hybrid

K7826	TCAGAGGAGGACCTGCATATGACAGATGAAAACACTG	TCGACGGATCCCCGGGAATTCATCCCAGCAATTA	Yeast two hybrid
K7859	TCAGAGGAGGACCTGCATATGGAGGAGGACTCAG	TCGACGGATCCCCGGGAATTCGTGTAGTATGACTG	Yeast two hybrid
K7860	TCAGAGGAGGACCTGCATATGAGTCTACCTATTGC	TCGACGGATCCCCGGGAATTCTTCCATTATATTTG	Yeast two hybrid
K4319	CGCGGATCCCAATGAGCAAAAACCCCATTGA	CCGCTCGAGGCCGCTCGAGGCCGCTCGAGG	Yeast two hybrid
K7222	GTACCAGATTACGCTCATATGATGAGCAAAAACCCCA	ATGCCCACCCGGGTGGAATTCCTCTTGGCTCTTGTAAAG	Yeast two hybrid
K7223	GTACCAGATTACGCTCATATGCAAGAAGCAGAATCGCGGCCT	ATGCCCACCCGGGTGGAATTCTCAAATTCGAACCGGT	Yeast two hybrid
K7224	GTACCAGATTACGCTCATATGATTCAAGCGCACAATGGGT	ATGCCCACCCGGGTGGAATTCTTTCCACACGTAAACATAGGA	Yeast two hybrid
K7795	GTACCAGATTACGCTCATATGCTTTACAAGAGCCAAGAG	ATGCCCACCCGGGTGGAATTCAACTACCCTCCATACATG	Yeast two hybrid
K7796	GTACCAGATTACGCTCATATGGATAAACCTTTTTGTTC	ATGCCCACCCGGGTGGAATTCGTCCCAAAGCCTCACT	Yeast two hybrid
K7797	GTACCAGATTACGCTCATATGAAGACTTGTTTGAGAATA	ATGCCCACCCGGGTGGAATTCGCTCCATATGCGAAC	Yeast two hybrid
K4320	GGGAATTCCATATGATGGAGAATTCAACTCAAG	CGCGGATCCAACTTTGAGAAGCTGCATTTTG	Yeast two hybrid
K4321	GGGAATTCCATATGATGGCCGCTAGCAGCGATCCT	CGCGGATCCTACCCTGAGAATCTGAAGC	Yeast two hybrid
K4322	GGGAATTCCATATGATGGAGAATTCAACTCAAG	CGCGGATCCAACTTTGAGAAGCTGCAATTTG	Yeast two hybrid
K4323	CCGGAATTCATGACGGCCACCAGCGATCCG	TCCCCCGGGTACCCTTAGAATCTGAAGC	Yeast two hybrid
K4324	GGGAATTCCATATGATGGATAATTCAGCTCCA	CGCGGATCCAACTCTAAGGAGCTGCA	Yeast two hybrid

K4014	GGGGTACCATGCTGAGCAAAATGCGGCCACCGT	TCCCCCGGGTCCCAACACAGGCGAACCACAT	Subcellular localization
K4015	GGGGTACCATGCTGAGCAAAATGCGGCCACCGT	TCCCCCGGGCTCACGGACCAACCTGGCTTC	Subcellular localization
K7933	ATTTACGAACGATAGGGTACCATGAGCAAAAACCCCATTG	GCTCACCATGGATCCGTCGACAATTCGAACCGGCAATC	Subcellular localization
K9052	ATTTACGAACGATAGGGTACCATGAGCAAAAACCCCATTGA	GCCCTTGCTCACCATGGATCCCTCTTGGCTCTTGTAAAGA	Subcellular localization
K9053	ATTTACGAACGATAGGGTACCATGCAAGAAGCAGAATCGCGGCCT	GCCCTTGCTCACCATGGATCCCCCCCGGGAATTCGAACCGGT	Subcellular localization
K9054	ATTTACGAACGATAGGGTACCATGATTCAAGCGCACAATGGGT	GCCCTTGCTCACCATGGATCCTTTCCACACGTAAACATA	Subcellular localization
K5322	GAGAACACGGGGGACTCTAGAATGCTGAGCAAAATGCGGCCA	GACAGTACTATCGATGGATCCTCCCAACACAGGCGAACCA	BiFC
K5323	GAGAACACGGGGGACTCTAGAATGCTGAGCAAAATGCGGCCA	GACAGTACTATCGATGGATCCCTCACGGACCAACCTGGC	BiFC
K5324	GAGAACACGGGGGACTCTAGAATGAGCAAAAACCCCATTG	GACAGTACTATCGATGGATCCAATTCGAACCGGCAATC	BiFC
K5412	CCAGCAGCATCCGCAGAATTCATGCTGAGCAAAATGC	ATGGTGGCTAGCGCTGAATTCTCCCAACACAGGCGAACCA	In vitro pull down
K5413	CCAGCAGCATCCGCAGAATTCATGCTGAGCAAAATGC	ATGGTGGCTAGCGCTGAATTCCTCACGGACCAACCT	In vitro pull down
K5414	TAAGAAGGAGATATACATATGATGAGCAAAAACCCCA	TCGTCGTCGGTACCCAGATCTAATTCGAACCGGCAAT	In vitro pull down
K7506	TCAGAGGAGGACCTGCATATGATGAGCAA	TCGACGGATCCCCGGGAATTCCTCTTGGCTCTTGTAAAG	Trans activation

	AAACCCCA		assay
K7507	TCAGAGGAGGACCTGCATATGATTCAAGCGCACAATGGGT	TCGACGGATCCCCGGGAATTCTTTCCACACGTAAACAT	Trans activation assay
K7508	TCAGAGGAGGACCTGCATATGCAAGAAGCAGAATCGCGGCC	TCGACGGATCCCCGGGAATTCCCCCCGGGAATTCGAA	Trans activation assay
K9266	CATCTCCCACACAAAGACATCA	TGGGCAGAGGAACAAAGAAAG	TA clone
K9269	AATCCCCACACCCTACGG	GCTGGAACGGGTAACTGG	TA clone
K9273	TTCCCCTTCACTTCTCCCAC	CCCCCAACACTTTGATGCTA	TA clone
K9038	ACCTAAAACACTCGTATGCTCAAA	TCAGACGAAGGAGTAGTTGAGTTG	TA clone
K9167	GAGAACACGGGGGACTCTAGAATGCTGAGCAAAATGCGGCCA	GGACTGACCACCCGGGGATCCTCCCAACACAGGCGAACCA	Vector construct
K9168	GAGAACACGGGGGACTCTAGAATGCTGAGCAAAATGCGGCCA	GGACTGACCACCCGGGGATCCCTCACGGACCAACCTGGC	Vector construct
K9169	GAGAACACGGGGGACTCTAGAATGAGCAAAAACCCCATTG	GGACTGACCACCCGGGGATCCAATTCGAACCGGCAATCC	Vector construct
K9180	GTTAATTAAGAATTCGAGCTCCTGATTCTTTTTGACTGAT	GTTGTGTTGAGAATTCTCGAGGACTTTTCGTTTTTTTTTTGGTGGCATGAATTCAGGGTAAAAT	Vector construct
K9181	GTTAATTAAGAATTCGAGCTCAAAATTTTCATTTAAGTTA	GTTGTGTTGAGAATTCTCGAGGACTTTTCGTTTTTTTTTTGGTGGCATGCTTTCTTTATGAGCA	Vector construct
K9205	CTATAGGGCGAATTGGGTACCCTGATTCTTTTTGACTG	TGTTTTTGGCGTCTTCCATGGGAATTCAGGGTAAAATC	Vector construct
K9206	CTATAGGGCGAATTGGGTACCAAAATTTTCATTTAAGTTA	TGTTTTTGGCGTCTTCCATGGGCTTTCTTTATGAGCA	Vector construct
K9156	CTTGAATTCGAGCTCGGTACCCTGATTCTTTTTGACTG	GTCGACAGATCCCCGGGTACCGAATTCAGGGTAAAATC	Vector construct
K9157	CTTGAATTCGAGCTCGGTACCAAAATTTTCATTTAAGTTA	GTCGACAGATCCCCGGGTACCGCTTTCTTTATGAGCA	Vector construct
K6432	AACCCAAAGGCCAACAGAGA	AAGGTCACGTCCAGCAAGGT	qRT-PCR

K6433	CTGAATCTTCGCTTTCACGTTATC	GGGATGCAAATCTTCGTGAAAAC	qRT-PCR
K103	CTTACCACCCGCCCCACTA	GAAGGGAAACGAAGAGTTGAGGT	qRT-PCR
K9213	TAAGGTCGCCTACTATGCCG	AAAGGACCGATTCCGTTAGG	qRT-PCR
K9215	GGGGTGGAACAAGCAGGAC	CAGTGTCGGGAAGCGGATA	qRT-PCR
K9217	GGGGATGATACCCAGGCTCT	TTCGTAGCCAGCAGAGTAAACAC	qRT-PCR
K9219	TCCCCATAGTTCCTGCCACA	TTCAGCCCCAGCCGTGTA	qRT-PCR
K9221	CAATCATCCTCCTACACCCGA	GGCTCCTGGTCCGAGCTG	qRT-PCR

Table S2 Proteins interacting with GhMML4_D12 identified by Y2H Screening.

Putative protein type	Average frequency	Gene ID
Phosphate-induced protein 1	4	Gh_A13G0604
SAP domain RNSP1-SAP18 binding (RSB) motif	5	Gh_A02G1275
Cytochrome P450	4	Gh_A08G1098
Transducin/WD40 repeat-like superfamily protein	7	Gh_D01G0508
Heavy-metal-associated domain	3	Gh_D01G1640
Zinc finger, C3HC4 type (RING finger)	2	Gh_A12G0544
BES1/BZR1 plant transcription factor	2	Gh_A10G0312
Ubiquitin-like protein smt3	3	Gh_A07G0763
Short chain dehydrogenase	1	Gh_D07G2351
